# Modelling Soil Water Retention Using Support Vector Machines with Genetic Algorithm Optimisation

**DOI:** 10.1155/2014/740521

**Published:** 2014-03-17

**Authors:** Krzysztof Lamorski, Cezary Sławiński, Felix Moreno, Gyöngyi Barna, Wojciech Skierucha, José L. Arrue

**Affiliations:** ^1^Department of Metrology and Modelling of Agrophysical Processes, Institute of Agrophysics, Polish Academy of Sciences, Doświadczalna 4, 20-290 Lublin, Poland; ^2^Institute for Natural Resources and Agrobiology (IRNAS-CSIC), P.O. Box 1052, 41080 Sevilla, Spain; ^3^Department of Crop Production and Soil Science, Georgikon Faculty, University of Pannonia, Deák Ferenc Street 16, Keszthely, 8360, Hungary; ^4^Aula Dei Experimental Station (EEAD-CSIC), P.O. Box 13034, 50080 Zaragoza, Spain

## Abstract

This work presents point pedotransfer function (PTF) models of the soil water retention curve. The developed models allowed for estimation of the soil water content for the specified soil water potentials: –0.98, –3.10, –9.81, –31.02, –491.66, and –1554.78 kPa, based on the following soil characteristics: soil granulometric composition, total porosity, and bulk density. Support Vector Machines (SVM) methodology was used for model development. A new methodology for elaboration of retention function models is proposed. Alternative to previous attempts known from literature, the *ν*-SVM method was used for model development and the results were compared with the formerly used the C-SVM method. For the purpose of models' parameters search, genetic algorithms were used as an optimisation framework. A new form of the aim function used for models parameters search is proposed which allowed for development of models with better prediction capabilities. This new aim function avoids overestimation of models which is typically encountered when root mean squared error is used as an aim function. Elaborated models showed good agreement with measured soil water retention data. Achieved coefficients of determination values were in the range 0.67–0.92. Studies demonstrated usability of *ν*-SVM methodology together with genetic algorithm optimisation for retention modelling which gave better performing models than other tested approaches.

## 1. Introduction

Soil hydrologic parameters have great impact on soil water transport processes. The soil water retention curve and soil water hydraulic conductivity are required for an appropriate description of soil water phenomena, such as drainage, infiltration, or soil pollutant movement. The retention curve describes the relationship between soil water content and soil water potential and is especially important for hydrological modelling and agronomical practice as it determines soil water availability for plants.

Measurements give strict evaluation of hydraulic properties of soils. Unfortunately, measurement of the soil water retention curve is time consuming and requires specialised equipment. The classical pressure plate extractor technique [1] may be used to determine the soil water retention curve, or an alternative technique based on the dynamic simultaneous time-domain reflectometry soil water content and pressure head measurements [2].

Fortunately in many applications the hydraulic parameters can be estimated rather than measured. Pedotransfer functions (PTF) are commonly used in such circumstances [3] and allow for estimation of the retention curve or hydraulic conductivity based on easily measured soil characteristics. The most widely used soil characteristics for PTF development are granulometric composition, bulk density, and organic matter content.

The soil water retention curve basically may be modelled in two ways: indirectly (parametric PTFs) and directly (also known as point PTFs). The first method evaluates parameters of some models of the soil water retention curve, for instance, the Mualem-van Genuchten function parameters: *θ*_*s*_, *θ*_*r*_, *α*, and *n* [4]; the latter evaluates water content for a specified number of water potential values. Recently a new method of retention modelling has been developed, which directly estimates soil water content but is not limited in estimations to fixed set of soil water potentials [5]. This so-called pseudocontinuous approach allows for estimation of the soil water content for any potential value in the range from 0 kPa to model dependent minimum value.

There have been numerous attempts to develop PTFs for retention curve, utilising a wide set of mathematical methods. Regression modelling is a widely used tool for PTF model development. Some methods rely simply on granulometric distribution as input parameters [6], and others also use soil bulk density [7–9]. The mean weight diameter of soil particles, a granulometric composition dependent parameter, is used by some authors [7] too. Soil content of organic carbon is another widely used parameter [9–11] for development of PTFs. Other models [12] additionally use soil water content for specified potentials, for example, −33 kPa and −1500 kPa.

Artificial neural networks (ANNs) are another technique often used for developing PTFs. Feed forward or radial basis neural networks allow for estimation of some set of output parameters based on knowledge of input parameters. ANNs were extensively used as a tool for PTF developments [13–17]. Unfortunately, there are some problems specific to artificial neural network modelling such as a tendency to stacking in local minima of mean square error hyperplane during ANN training process [18] or difficulties with appropriate choice of ANN architecture which causes overfitting of ANN to training data. The partial solution to this problem is based on bootstrap averaging, which averages the predictions of many ANNs trained on randomly modified input data [19].

Recently another mathematical tool, the Support Vector Machine (SVM), has been used for PTF modelling. This technique resolves typical problems for ANN-based PTFs development. There have been attempts of SVM usage for PTF development in a direct manner [20] and in parametric form [21] where Mualem-van Genuchten parameters were estimated. Both of the SVM models were compared with ANN-based counterparts and showed better performance in water retention modelling.

In this work we focused on the development of the point PTFs for estimation of the soil water retention curve using SVM and on some of its methodological aspects. We investigated whether the newer *ν*-SVM method, not used before in PTF studies, was applicable and possibly better for this purpose than typically used *C*-SVM method. For the purpose of automated models' parameters search genetic algorithms were used as an optimisation framework. A new form of the aim function used for models' parameters search is proposed, which allows for better selection of models' parameters. As a result more accurate PTF models may be developed.

## 2. Material and Methods

### 2.1. Soil Datasets

Soil dataset used in this study was an extract from the Soil Profiles Bank of the Polish Mineral Soils database [22] and contained 639 soil samples, taken from 290 different soil profiles. Soil samples were collected from three horizons for most soil profiles. Undisturbed samples were collected into the metal cylinders of volume 100 cm^3^ and diameter 5 cm, and then basic soil parameters were analysed. The following soil parameters were extracted from the soil database for the purposes of this work: soil water content for various six soil water potential values: −0.98 kPa, −3.10 kPa, −9.81 kPa, −31.02 kPa, −491.66 kPa, and −1554.78 kPa; particle size distribution; total porosity; and bulk density. Particle size distribution was determined for the following fractions: clay <0.002 mm, silt 0.002–0.05 mm, and sand 0.05–1 mm. Basic statistics of soil dataset used in this study are presented in Table 1.

### 2.2. SVM Methodology

SVM is one of the classes of soft-computing techniques [23]. Originally SVM was developed for solving classification problems; then its usage has been extended to regression-type applications for function estimation [24]. SVMs used for regression modelling estimate one output variable based on a set of input variables. As being supervised learning method SVM uses training dataset for model development. Elaborated model reproduces input-output relationship present in the training dataset and is capable of making estimations based on arbitrary input variables.

From the user's perspective, SVM model development consists of the following steps:

- training and testing datasets preparation,
- SVM model selection (*C*-SVM or *ν*-SVM),
- selection of the kernel function,
- selection of the SVM model and kernel function parameters,
- model learning using training dataset,
- validation of the model against training dataset,
- validation of developed model against testing dataset.

If one is optimising model's parameters the steps (d) and (e) may be repeated until best parameters will be found.

The training dataset $D=\left\{\left[\overset{-}{x}\left(i\right),y\left(i\right)\right]\in {\mathbb{R}}^{N}\times \mathbb{R},i=1,\ldots ,l\right\}$ consists of pairs where many input values are mapped to one output response value. Training data which are used for model development are specific to applications and the training dataset has to be representative for modelled problem. Quality of these data impacts on model generalisation capability and its ability for making accurate predictions.

There are two different types of SVM algorithms used in regression modelling: *C*-SVM and *ν*-SVM. The SVM methodology originally introduced by Vapnik is currently known as *C*-SVM or SVM Type 1 models. These models have two adjustable parameters which influence their behaviour: *C*—the so-called penalty parameter—and *ε*—insensitivity zone. *C* determines the mutual relationship between the training error and the model complexity. An increase in *C* causes penalising of larger errors, which leads to decreasing of approximation error. Insensitivity zone *ε* describes the tolerance for training error in the SVM model: a decrease in *ε* leads to strict fitting to training data, which may cause overfitting and decrease the model generalisation properties. SVM models with lower *ε* values use a larger number of support vectors.

Support vectors are selected data records taken from the training dataset. Which data records taken from the training dataset are support vectors is decided by SVM algorithm during model training phase. Support vectors are vital part of the developed model as they are used further by the SVM algorithm for making model estimations.

One of the most important properties of SVM regression models is the ability to generalise, which allows for appropriate predictions from previously unseen input data. The technical criterion which stands behind this requisite is a limit set on the number of support vectors to about half of all vectors in the training dataset [25].

If a SVM model with arbitrarily chosen support vectors percentage is required, it is convenient to use another SVM model formulation known as *ν*-SVM or SVM Type 2 models [26]. In this type of SVM models instead of *ε* another model parameter *ν* ∈ [0,1] is used, which is utilised for internal trading off *ε* between the model accuracy for training data and the model complexity (number of support vectors), which influences the model generalisation properties. Two parameters in *ν*-SVM regression models are used: *C* and *ν*. Formally, the *ν* parameter expresses the desired number of SVs and is a lower bound on the fraction of support vectors [26]. In *C*-SVM models, the number of support vectors participating in model formulation is indirectly related to the model parameters *C* and *ε* while in *ν*-SVM models it is connected with the parameter *ν* itself.

Kernel function is an important part of SVM model. It is used internally in the SVM algorithm to map the input parameters to highly dimensional feature space used in the algorithm internal computations. Thanks to nonlinear kernel functions SVM algorithm allows for estimations where dependence between estimated output variable and input variables is highly nonlinear. There are some commonly used kernel functions: linear $K\left({\overset{-}{x}}^{i},{\overset{-}{x}}^{j}\right)={\overset{-}{x}}^{i}\cdot {\overset{-}{x}}^{j}$, polynomial $K\left({\overset{-}{x}}^{i},{\overset{-}{x}}^{j}\right)={\left(\gamma \left({\overset{-}{x}}^{i}\cdot {\overset{-}{x}}^{j}\right)+a\right)}^{d}$, and radial basis $K\left({\overset{-}{x}}^{i},{\overset{-}{x}}^{j}\right)=e^{-\gamma {||{\overset{-}{x}}^{i}-{\overset{-}{x}}^{j}||}^{2}}$. The most often used kernel function in SVM regression modelling is radial basis kernel function. The linear kernel function is in fact a special case of a polynomial kernel function with fixed parameters: *γ* = 1, *a* = 0, and *d* = 1.

The main purpose of SVM model development is to select proper support vectors from training dataset. Proper selection of support vectors has an impact on model performance and the ability for generalisation. The SVM model's parameters together with kernel function parameters (if any) have to be thoroughly chosen while model building phase.

### 2.3. Genetic Algorithm Parameters Optimisation

SVM models depend on parameters which must be adjusted. Different types of models tested use different sets of parameters: SVM cost (*C*), intensive zone width (*ε*), and parameter (*γ*) of the radial basis kernel function.  Table 2 summarizes types of SVM models, kernel functions used, models parameters, and names of the models used for the reference in this paper.

Different models depend on different sets of parameters. The values of these parameters may be selected arbitrarily or determined using some kind of universal, developer independent procedure. In fact the determination of SVM model parameters is an optimisation problem and typical methods for such tasks may be used. Leave-one-out method [27] has been used in previous work, Lamorski et al. [20], but it is extremely computationally demanding. A simple search for an optimal solution on a grid of possible parameter values is another method commonly used [21]. Unfortunately grid search method does not test whole space of possible parameters values as it is limited to arbitrarily chosen fixed combinations of parameters. As a result nonoptimal models' parameters may be chosen. Some optimisation algorithms could be used instead.

Genetic algorithms (GA) with elitism were used in the present study for searching the optimal values of model parameters. GA is a technique used among others for optimisation purposes [28] and is especially well suited for applications where the aim function is not differentiable and may have local minima. None of the classical optimisation methods such as the Nelder-Mead downhill simplex method or gradient-based methods may be used successfully in such circumstances.

Parameters values searched for an optimal solution using GA were defined by the following bands: 1 < *C* < 1200, 0.0001 < *ε* < 1, and 0.00001 < *γ* < 10.

Genetic algorithm operation is controlled by two main parameters: population size and a number of GA iterations. The other vital GA parameters are elitism percentage and mutation chance. The elitism percentage used in our study was 0.2, and mutation chance was 0.02.

Population size determines the number of points in the space of input parameters, where the model performance is evaluated. A population size of 100 was used for searching of the parameters in that study.

### 2.4. Model Formulation

SVM was used for model development. The soil database was randomly split into two subsets: training dataset (414 samples) and testing dataset (225 samples). SVM models were built using the training dataset and tested against the test dataset. Test datasets were not used for model development at any stage, except for final model testing and validation.

Correlation analysis was performed on the soil dataset and used for model elaboration. The analysis of this data led to selection of the input parameters for developed models: sand fraction, clay fraction, total porosity, and bulk density.

Due to the fact that SVM regression models allow for estimation of only one output parameter for a given set of input parameters, six different SVM models were developed, one for each value of the soil water potential in which water content was evaluated.

The *k*-fold approach [19] was used for model elaboration, which allowed for a cross validation during the training phase of model development. The training dataset was randomly divided into ten distinct equinumerous subsets, for the purpose of the *k*-fold method.

The developed models' returned value, which was an average output from 10 different submodels, resulted from the *k*-fold method. This submodel was trained on the joined-together nine subsets (373 soil samples). The remaining tenth subset was used for cross validation purposes and was rotated for each of the ten submodels elaborated. The *k*-fold method allowed for estimation of variance of evaluated properties.

One of the major decisions made during SVM model development is to choose an appropriate kernel function. Previous attempts using SVM for retention curve modelling have applied the radial basis kernel function [20, 21]. In the present study we wished to investigate the influence of the kernel function on PTF model performance. We tested two kernel functions: linear kernel and radial basis kernel. The advantage of the linear kernel function over the radial basis kernel is the reduced number of model parameters. The radial basis kernel function introduces an additional parameter, *γ*, to the model, while the linear kernel function does not depend on any additional parameters.

One of the expected PTF model features is the ability for generalisation—when a model predicts correctly the results for previously unseen input data. The technical criterion in SVM model formulation that stands behind this model feature is the number of support vectors used for model formulation [25]. In commonly used *C*-SVM models there is no direct influence on the number of support vectors, which depend implicitly on other model parameters. The objective of the present study was to compare two classes of PTFs: *ν*-SVM based (with a fixed value of *ν*) and *C*-SVM. The parameter *ν*, in *ν*-SVM based models, explicitly determines expected percentage of SVs used in the model formulation. The model performance is checked with a theoretically optimal 50% of SVs, so the value of *ν* is fixed at 0.5.

### 2.5. Model Performance Criteria

Some kind of model performance criteria is needed in SVM model development for validation purposes. Typically root mean square error (RMSE) and the coefficient of determination (*R* ^2^) are used:

(1) $$\begin{array}{c} \text{RMSE}=\sqrt{\frac{1}{N}\overset{N}{\underset{i=1}{\sum }}{\left(y_{p}-y_{m}\right)}^{2}}, \end{array}$$

(2) $$\begin{array}{c} R^{2}=1-\frac{\sum_{i=1}^{N}{\left(y_{p}-y_{m}\right)}^{2}}{\sum_{i=1}^{N}{\left(y_{m}-{\hat{y}}_{m}\right)}^{2}}, \end{array}$$

where *N* is the number of data analysed, *y*_*p*_ is a value approximated by the model, *y*_*m*_ is the “true” measured value, and ${\hat{y}}_{m}$ is the mean of the measured values. In ideal conditions, when the values approximated by the model equal the measured ones, then *R* ^2^ = 1 and RMSE = 0.

## 3. Results and Discussion

### 3.1. Overfitting and the Radial Basis Kernel Function

RMSE (1) and *R* ^2^ (2) are widely used model performance criteria for PFT development. Model parameters are adjusted to minimise the RMSE for the training dataset. Usually a model which minimises RMSE for the training dataset also has a small RMSE for the test dataset—if so, the model has good generalisation properties.

One of the main machine learning paradigms states that only the training dataset is used for model development. On the other hand, the testing dataset is used only at the very last step, to check how developed model performs for unseen previously data. A model minimising RMSE between estimations and measured values from testing dataset is considered to be the best model.

At the initial stage of the model development RMSE was used as the aim function and the GAs were used for seeking the optimal model's parameters. For both models utilising the linear kernel function, the* C-linear* and* nu-linear*, usage of RMSE as the aim function was a successful strategy. In that case the GA was able to find optimal model parameters.

However, when the radial basis kernel function was used, GA optimisation led to overfitting regardless of the type of the SVM method used. Very low RMSE values (<0.001) for the training dataset were achieved; however, for the test dataset the RMSE was high. The generalisation capability of these developed models was very poor, and the number of support vectors was close to the number of all training vectors—which is an indicator of overfitting of the model. This phenomenon was caused by a too high value of the radial basis kernel function's parameter *γ* determined by GA. Thus the GA algorithm was choosing the highest available value of *γ*, that is, an upper limit of the range of possible *γ* parameter values.

The SVM models seem to be especially sensitive to the value of *γ* parameter of the radial basis kernel function. The increasing value of *γ* leads to an increased number of support vectors in the model, which degrades its generalisation capabilities. When RMSE was used as the aim function, GAs chose optimal values of parameters which minimised RMSE for the training dataset. But for these parameters RMSE was relatively high for the testing dataset, so parameters were not acceptable from the point of view of model generalisation capabilities. In fact using RMSE as the aim function together with genetic algorithms optimisation led to development of models which were not optimal when radial basis kernel function was used.

**Figure 1:**
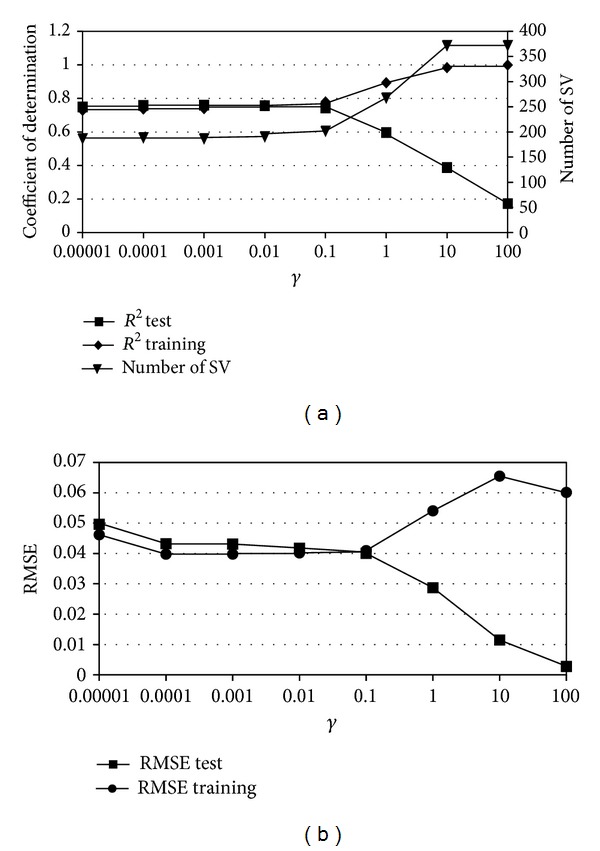
Example of the overfitting phenomenon for the radial basis kernel based *ν*-SVM model.

**Figure 2:**
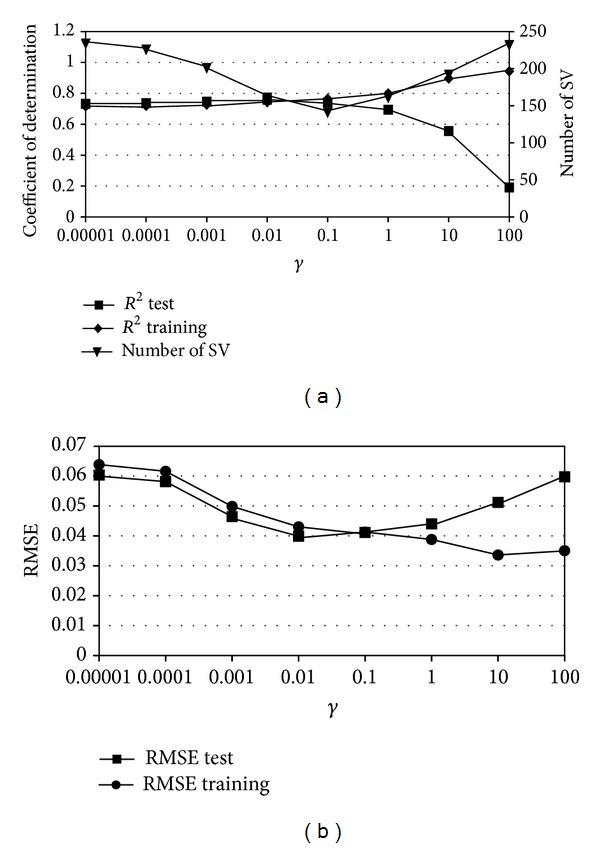
Example of the overfitting phenomenon for the radial basis kernel based *C*-SVM model.

An example of overfitting phenomenon for the *ν*-SVM model, which evaluates the value of water content for the soil water potential of −0.98 kPa, is given in Figure 1.  Figure 1(a) shows the dependence between *R* ^2^ and *γ*, calculated for the training dataset (*R* ^2^ train) and the testing dataset (*R* ^2^ test). The other *ν*-SVM model parameters are as follows: *C* = 100 and *ν* = 0.5. For values of *γ* > 0.1, the value of *R* ^2^ for the training dataset increased until an unrealistic value *R* ^2^ = 1 (overfitting) was reached, while *R* ^2^ for testing dataset decreased greatly (i.e., lack of generalisation capabilities).  Figure 1(a) gives also dependence between the number of support vectors (number of SV) in the model and the value of parameter *γ*. It can be observed that for high values of *γ* the number of SV in the model reaches the maximum 373 (whole *k*-fold training dataset)—which is also the evidence of overtraining. Similarly (Figure 1(b)) RMSE for training dataset is decreasing for growing values of *γ* and RMSE for testing dataset is increasing.  Figure 2 shows similar dependence between parameter *γ* and models statistical characteristics RMSE and *R* ^2^ for *C*-SVM based model estimating water content for soil water potential −0.98 kPa.

To give better insight into models structure, sensitivity analysis was performed. For this purpose, the Morris [29] global sensitivity analysis method, modified according to Campolongo et al. [30], was used in its classical formulation: the one-step-at-a-time (OAT) approach. The outcome from sensitivity analysis was index μ*, which represents relative to other parameters the impact of tested model parameter on model outcome averaged over the whole parameters space. The model outcome which was used for the purpose of sensitivity analysis was a sum of model estimates made for all the records from training dataset.

**Figure 3:**
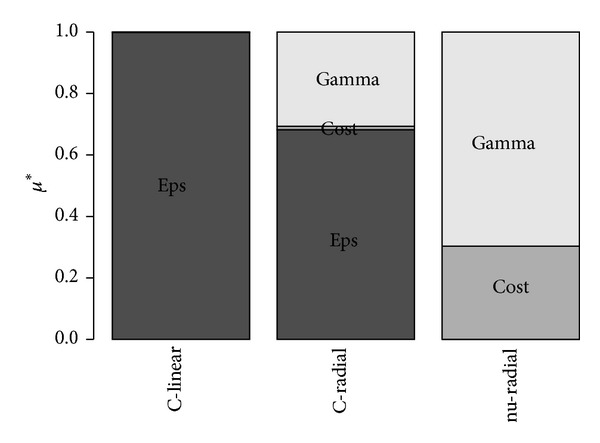
Morris global sensitivity analysis, scaled μ* indices determined for the SVM models estimating soil water content for the potential −0.98 kPa.

Figure 3 presents normalized values of sensitivity indices μ* determined for all models estimating water content for the soil water potential −0.98 kPa as an example. Results for model nu-linear are missing as this model depends on only one parameter *C*. What can be seen here is relatively low dependence of the model on the SVM cost parameter *C*. Model *C*-SVM utilizing linear kernel function (*C*-linear), which depends on *ε* and *C* parameters, is almost not sensitive to parameter *C*. The same for the *ν*-SVM linear kernel model, where the parameter *C* has low influence on model's outcome. Radial basis kernel function *ν*-SVM model (nu-radial) depends on two parameters: *C* and *γ*, and the latter has much higher impact on models estimations.

### 3.2. Alternative Form of the Aim Function

Models for the other soil water potentials, both *C*-SVM and *ν*-SVM based, demonstrated the same behaviour (overfitting) when RMSE was used as the aim function and the radial basis kernel function was used. If some kind of automated model-parameter search method such as GA is to be used, then the use of RMSE as the aim function is discouraged because of the dominant influence of *γ* on its value.

An aim function is needed that will explicitly take into account both factors: the model performance criterion (e.g., RMSE) and the model generalisation capabilities. An alternative form of the aim function, instead of RMSE, is proposed in (3)

(3) Faim(RMSE,nSV) =1−e−RMSE2/(2σrmse2)e−(nSV−nSVexp⁡)2/(2σnSV2).

Equation (3) is dependent on two arguments: RMSE and the number of support vectors in the elaborated model (nSV). This formula mimics the normal bivariate distribution, with the parameters nSV_exp⁡_, *σ*_rmse_^2^, and *σ*_nSV_^2^ which are constants.

The proposed new aim function has a minimum at the point where RMSE = 0 and nSV = nSV_exp⁡_, both of these criteria have to be met in the minimum of this function. The parameter nSV_exp⁡_ is the number of support vectors expected in the developed model. The value of the parameter nSV_exp⁡_ should be equal to half of the total number of the input data in the training dataset [25, 26] to achieve an optimal nonovertrained model. In the present study, nSV_exp⁡_ = 187 as we have 373 records in each *k*-fold training dataset and we assumed theoretical ideal proportion (1/2) between the number of support vectors and the number of data in the training dataset.

**Figure 4:**
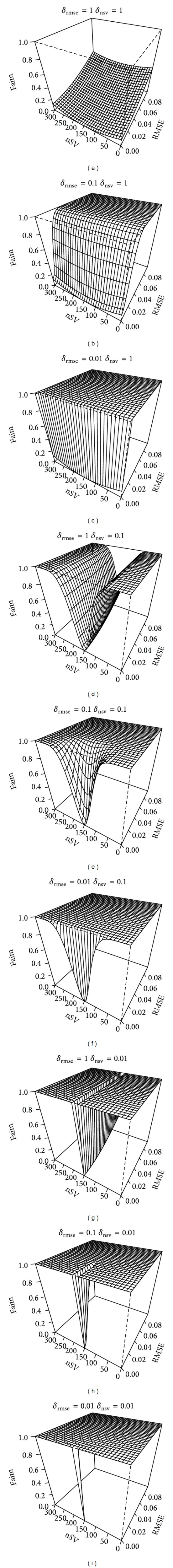
The shape of the aim function and its dependence on aim function parameters.

Constants *σ*_rmse_ and *σ*_nSV_ have the impact on the shape of the aim function and are connected with its slopes. The values of these parameters were selected based on the empirical basis, numerical tests, and analysis of their influence on the shape of the aim function (Figure 4). The parameter *σ*_rmse_ = *δ*_rmse_RMSE_max⁡_ was related to the RMSE_max⁡_, the maximum value of RMSE which could occur during SVM model parameter search procedure. In this study value of RMSE_max⁡_ was estimated altogether with sensitivity analysis of the models, but achieved values of RMSE_max⁡_ were pretty standard for point PTF developments and could be chosen arbitrarily.

Similarly, the parameter *σ*_nSV_ = *δ*_nSV_nSV_max⁡_ was related to the nSV_max⁡_, the total number of the records in the training dataset. In cases of the parameter *σ*_rmse_ the appropriate value of *δ*_rmse_ = 1 while in case of the parameter *σ*_nSV_ the good value of *δ*_nSV_ = 0.1. Figure 4 presents the shape of the aim function in relation to the values of the *δ*_rmse_ and *δ*_nSV_ parameters.

Instead of simple RMSE formula new aim function (3) was used in conjunction with GA optimisation techniques for searching for models' parameters. For *ν*-SVM based models (nu-radial) an optimal solution was found, and both criteria were reached: RMSE was low and an optimal number of support vectors were selected. For *C*-SVM based models (*C*-radial) the results were much better than found previously when RMSE was used as an aim function but were still inadequate.

### 3.3. PTF Model Performance

Finally eight types of PTF models were developed, one for each value of the soil water potential for which water content was estimated. These models utilised *C*-SVM and *ν*-SVM based modelling and both kernel functions: radial and linear. Two types of aim function were used together with GA for models development. Comparison of results for these eight models presents Table 3.

Table 3 summarises performance indices of elaborated models: number of support vectors, RMSE, and *R* ^2^; all calculated for the training and testing dataset. Values of statistical indices RMSE and *R* ^2^ were calculated from comparisons of evaluated model values against measurements of soil water content for specified values of soil water potential.

The ability of the model to predict correctly previously unseen data is very important, so model's results for testing dataset are most interesting.

In case of *ν*-SVM models based on the radial basis kernel function, the use of the aim function in a new form, proposed in this paper, allowed for selection of much better model's parameters. As a result developed models are not overtrained and perform much better than models which were developed using RMSE as the aim function.

We may state that the best combination of SVM algorithm used kernel function and method of models parameter search was radial basis kernel function based *ν*-SVM based model trained using the aim function in the form of (3).

Newly proposed aim function increased also substantially results achieved in case of *C*-SVM radial basis kernel function based models, but in this case the results are not good enough to use these models anyway. Other models perform better, even linear kernel based.

We can see (Table 3) that when linear kernel is used, there is really no difference between models developed using different methodology. Regardless the SVM algorithm type used (*ν*-SVM or *C*-SVM) or aim function used for GA parameter search the results achieved by the models are the same. The small differences in RMSE (0.0495–0.0517) occurring for the soil water potential −491.66 kPa in case of *C*-linear model are not important from practical point of view of PTF usage.

This information, together with the observation that the models are almost not sensitive to the value of the parameter *C* (which is the only parameter in case of *ν*-SVM model), gives the conclusion that PTF models based on *ν*-SVM algorithm together with linear kernel function may be constructed without any optimisation of the value of the *C* parameter. The general rules for selection of the value of *C* parameter [31] may be used instead.

When results for the testing dataset were considered, the conclusion was that the nu-radial model based on the *ν*-SVM method and the radial basis kernel function was the model of first choice for further PTF evaluations. However, the nu-linear model was also very appealing, due to having accuracy not much lower than that of the nu-radial counterpart while having only one model parameter, which simplifies substantially model development. One note has to be made regarding this statement. It is known from the literature of SVM kernel algorithms that linear kernel function may be considered as the alternative to radial basis kernel only for big training datasets. In case of smaller training datasets radial basis kernel function should be used. Results obtained here suggest that in our case the number of soil data used form model development was almost numerous enough (373 records) to use linear kernel function for model development.

## 4. Conclusions

SVM methodology was successfully applied to water retention modelling. Elaborated point PTF models used sand fraction, clay fraction, total porosity, and bulk density as the input parameters.

Thenewly proposed *ν*-SVM based retention models, with a fixed value of *ν* = 0.5, showed better performance than *C*-SVM based models.

Proposed, new form of the aim function (3) used for searching of the model's parameters allows for development of better models in case of radial basis kernel based models.

Results of this study showed that the *ν*-SVM method is suitable for the development of PTF models for retention curve approximation. The advantage of using this method is a limited number of model parameters in comparison with *C*-SVM methodology.

The investigated linear kernel function may be used successfully instead of the radial basis function, for point PTF developments. This kind of kernel function allows for reduction of the model parameters by one compared with the radial basis kernel function and also for simplified model development.

## Figures and Tables

**Table 1:** Basic statistics of the soil dataset.

Variable name	Mean	Standard deviation	Minimum	Maximum
Sand percentage	63.7	25.5	3.0	100.0
Silt percentage	25.9	18.8	0.0	81.0
Clay percentage	10.4	12.6	0.0	73.0
Bulk density (g/cm^3^)	1.65	0.19	0.98	2.17
Total porosity	0.41	0.0651	0.223	0.636

**Table 2:** Models and their parameters.

Model name abbreviation	SVM method	Kernel function	Model parameters	Number of model parameters
C-radial	*C*-SVM	Radial	C, *ε*, *γ*	3
C-linear	*C*-SVM	Linear	C, *ε*	2
nu-radial	*ν*-SVM	Radial	C, *γ*	2
nu-linear	*ν*-SVM	Linear	C	1

**Table 3:** Developed models performance comparisons.

Potential (kPa)	Model type	Number of SV		Training dataset				Testing dataset			
				*R* ^2^		RMSE		*R* ^2^		RMSE	
		Aim 1	Aim 2	Aim 1	Aim 2	Aim 1	Aim 2	Aim 1	Aim 2	Aim 1	Aim 2
−0.98	C-linear	137.2	179.1	0.92	0.92	0.0184	0.0185	0.90	0.90	0.0194	0.0193
		(13.71)	(6.66)	(0.006)	(0.006)	(0.0005)	(0.0005)				
	nu-linear	189.8	189.8	0.92	0.92	0.0186	0.0186	0.90	0.90	0.0194	0.0194
		(1.99)	(1.81)	(0.006)	(0.006)	(0.0005)	(0.0005)				
	C-radial	359.5	187.1	0.99	0.97	0.0051	0.0116	0.60	0.77	0.0404	0.0293
		(5.38)	(1.73)	(0.001)	(0.001)	(0.0003)	(0.0002)				
	nu-radial	372.3	198.4	0.99	0.94	0.0052	0.0160	0.59	0.92	0.0414	0.0175
		(3.13)	(4.58)	(0.001)	(0.006)	(0.0003)	(0.0005)				

−3.1	C-linear	92.5	117.7	0.62	0.62	0.0485	0.0497	0.67	0.68	0.0384	0.0391
		(7.11)	(85.18)	(0.010)	(0.008)	(0.0008)	(0.0011)				
	nu-linear	189.1	189.2	0.61	0.61	0.0497	0.0497	0.68	0.68	0.0363	0.0363
		(1.45)	(1.81)	(0.011)	(0.011)	(0.0009)	(0.0009)				
	C-radial	352.1	187.4	0.92	0.84	0.0225	0.0312	0.23	0.48	0.0735	0.0492
		(10.79)	(1.35)	(0.007)	(0.012)	(0.0008)	(0.0009)				
	nu-radial	372.4	194.5	0.92	0.68	0.0227	0.0448	0.22	0.70	0.0770	0.0348
		(2.80)	(2.88)	(0.007)	(0.018)	(0.0007)	(0.0010)				

−9.81	C-linear	134.0	133.7	0.76	0.75	0.0516	0.0529	0.72	0.72	0.0497	0.0499
		(14.34)	(111.77)	(0.004)	(0.011)	(0.0007)	(0.0016)				
	nu-linear	189.7	189.7	0.76	0.76	0.0517	0.0517	0.72	0.72	0.0498	0.0498
		(1.49)	(1.77)	(0.004)	(0.004)	(0.0007)	(0.0007)				
	C-radial	360.2	187.7	0.98	0.93	0.0151	0.0282	0.35	0.63	0.0959	0.0577
		(8.46)	(1.77)	(0.003)	(0.003)	(0.0014)	(0.0006)				
	nu-radial	372.4	194.8	0.98	0.85	0.0152	0.0416	0.33	0.82	0.0985	0.0395
		(2.95)	(2.49)	(0.004)	(0.007)	(0.0015)	(0.0010)				

−31.02	C-linear	141.1	123.1	0.77	0.77	0.0516	0.0528	0.71	0.70	0.0528	0.0532
		(6.90)	(102.16)	(0.004)	(0.008)	(0.0006)	(0.0012)				
	nu-linear	190.0	189.9	0.77	0.77	0.0522	0.0522	0.71	0.71	0.0532	0.0532
		(2.05)	(2.42)	(0.004)	(0.004)	(0.0006)	(0.0006)				
	C-radial	351.2	187.8	0.99	0.93	0.0123	0.0292	0.36	0.67	0.0957	0.0554
		(11.31)	(2.20)	(0.001)	(0.005)	(0.0007)	(0.0009)				
	nu-radial	372.3	195.6	0.99	0.85	0.0126	0.0419	0.34	0.80	0.0998	0.0436
		(3.06)	(4.03)	(0.002)	(0.007)	(0.0009)	(0.0007)				

−491.66	C-linear	109.0	80.8	0.72	0.71	0.0502	0.0518	0.67	0.65	0.0495	0.0517
		(17.95)	(54.42)	(0.008)	(0.016)	(0.0013)	(0.0023)				
	nu-linear	189.3	189.7	0.71	0.71	0.0508	0.0509	0.67	0.67	0.0493	0.0493
		(1.77)	(1.64)	(0.009)	(0.009)	(0.0015)	(0.0015)				
	C-radial	364.8	186.9	0.99	0.93	0.0091	0.0252	0.36	0.60	0.0875	0.0569
		(7.64)	(2.51)	(0.002)	(0.004)	(0.0011)	(0.0006)				
	nu-radial	372.5	202.1	0.99	0.80	0.0092	0.0421	0.35	0.70	0.0890	0.0475
		(3.10)	(8.35)	(0.002)	(0.020)	(0.0011)	(0.0026)				

−1554.78	C-linear	92.7	136.1	0.69	0.68	0.0459	0.0470	0.63	0.63	0.0468	0.0465
		(21.16)	(77.41)	(0.012)	(0.013)	(0.0016)	(0.0014)				
	nu-linear	189.4	189.7	0.68	0.68	0.0464	0.0464	0.64	0.64	0.0463	0.0463
		(1.90)	(2.21)	(0.014)	(0.014)	(0.0017)	(0.0017)				
	C-radial	364.6	187.7	0.99	0.92	0.0093	0.0232	0.32	0.58	0.0794	0.0511
		(5.62)	(2.21)	(0.002)	(0.003)	(0.0008)	(0.0008)				
	nu-radial	372.5	199.9	0.99	0.77	0.0094	0.0396	0.32	0.67	0.0805	0.0446
		(2.95)	(7.49)	(0.002)	(0.025)	(0.0008)	(0.0026)				

The presented values are averages of the other ten *k*-fold submodels. In the case of the number of support vectors, RMSE and *R* ^2^ for the training dataset and values in brackets are standard deviations. Columns described by “aim 1” present data for models developed using RMSE as the aim function. Label “aim 2” is linked with models developed using proposed new form of the aim function.

## Abbreviations

ANN: Artificial neural network
SVM: Support Vector Machines
SV: Support vector
PTF: Pedotransfer function
SWRC: Soil water retention curve
TDR: Time-domain reflectometry.
